# Differential Evolvability Along Lines of Least Resistance of Upper and Lower Molars in Island House Mice

**DOI:** 10.1371/journal.pone.0018951

**Published:** 2011-05-11

**Authors:** Sabrina Renaud, Sophie Pantalacci, Jean-Christophe Auffray

**Affiliations:** 1 Laboratoire de Biométrie et Biologie Evolutive, Université Lyon 1, CNRS, Villeurbanne, France; 2 Molecular Zoology Team, Institut de Génomique Fonctionnelle de Lyon, Université de Lyon, CNRS, Ecole Normale Supérieure de Lyon, Lyon, France; 3 Institut des Sciences de l'Evolution, Université Montpellier 2, CNRS, Montpellier, France; University College London, United Kingdom

## Abstract

Variation within a population is a key feature in evolution, because it can increase or impede response to selection, depending on whether or not the intrapopulational variance is correlated to the change under selection. Hence, main directions of genetic variance have been proposed to constitute “lines of least resistance to evolution” along which evolution would be facilitated. Yet, the screening of selection occurs at the phenotypic level, and the phenotypic variance is not only the product of the underlying genetic variance, but also of developmental processes. It is thus a key issue for interpreting short and long term evolutionary patterns to identify whether main directions of phenotypic variance indeed constitute direction of facilitated evolution, and whether this is favored by developmental processes preferably generating certain phenotypes. We tackled these questions by a morphometric quantification of the directions of variance, compared to the direction of evolution of the first upper and lower molars of wild continental and insular house mice. The main phenotypic variance indeed appeared as channeling evolution between populations. The upper molar emerged as highly evolvable, because a strong allometric component contributed to its variance. This allometric relationship drove a repeated but independent evolution of a peculiar upper molar shape whenever size increased. This repeated evolution, together with knowledge about the molar development, suggest that the main direction of phenotypic variance correspond here to a “line of least developmental resistance” along which evolution between population is channeled.

## Introduction

Variation within a population is a key feature in evolution, because it can increase or impede response to selection, depending on whether or not the intrapopulational variance is correlated to the change under selection [1], [2], [3]. Hence, main directions of genetic variance have been proposed to constitute “lines of least resistance to evolution” [4] along which evolution would be facilitated [5]. Yet, the screening of selection occurs at the phenotypic level, and the phenotypic variance is not only the product of the underlying genetic variance, but also of developmental processes. Recent advances in understanding the intricate interplay of development and evolution (“evo-devo”) brought evidences that some developmental processes favor the production of certain phenotypes (e.g. [6]), thus channeling the course of long-term evolution into preferred directions [7]. The main directions of phenotypic variance may thus point to “lines of least developmental resistance” to evolution. Focusing on how phenotypic variance can contribute to the course of evolution, and asking whether it express developmental properties, is thus a new and promising issue to the understanding of both short and long term evolution [7], [8], [9], [10].

We develop such an approach on mammalian dentition, and more specifically on the house mouse molars. It is a long running evolutionary issue how the complex, multicuspid mammalian dentition evolved in response to selective pressures related to feeding strategies, within constraints related to the phylogenetic history (e.g. [11], [12], [13], [14], [15]). Recent studies that revolutionized the conception of how dental phenotypic variation could be generated also focused on mammalian dentition by modeling tooth development (e.g. [6], [7], [16], [17]). One of the main results was the realization that large phenotypic differences could be the result of much simpler genetic differences than previously thought. For instance, small accessory cusps appeared to be easily produced by small differences in the inhibitory field surrounding the main forming cusps, thus explaining how populations can be polymorphic for this trait [6], [18].They suggested that developmental processes can channel long-term evolution, using a model of the molar development in the mouse to explain the diversification of molar proportions in related species [7].

**Figure 1:**
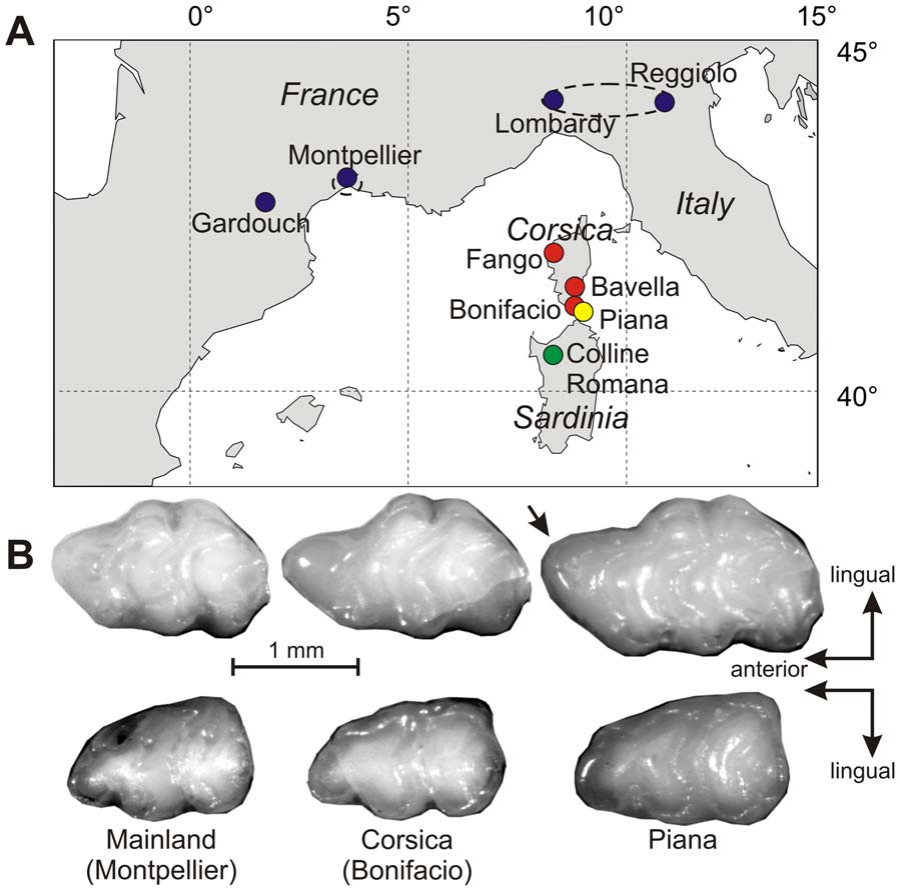
Sampling area and illustration of the tooth variation. (A) Localization of the sampling localities, with colors corresponding to the mainland and the different islands. Pooling of several localities in larger geographic groups is indicated by dotted lines. (B) Example of upper (top panel) and lower (bottom panel) molars of the house mouse (*Mus musculus domesticus*) in mainland Southern France (Montpellier), Corsica and the islet Piana. The arrow points to the prestyle on the first upper molar from Piana.

The house mouse dentition is a particularly relevant model. It combines a rich background on development and genetics of the teeth based on laboratory mice (e.g. [7], [19], [20]) and good knowledge of wild populations including numerous islands where marked morphological divergence has occurred in very short time [21], [22], [23]. In particular, Mediterranean island populations of the house mouse exhibit intriguing morphological variation of molar shape [23]. Especially the occurrence of an unusual furrow at the anterior periphery of the first upper molar (arrow on Fig. 1) suggests that it might correspond to the emergence of an additional cusp, a pattern of interest for developmental studies (e.g. [6], [18]).

Using these island populations as a model of divergence and populations from the nearby mainland as a reference, we quantified the size and shape of the first upper and lower molars, and investigated the patterns of intra-population variance and inter-population divergence. We then addressed the following issues:

- Does inter-population divergence occur following main directions of intra-population variation, validating their role as “line of least resistance” to evolution [4]?
- Can we relate the directions of main variation with developmental knowledge to explain and possibly predict from the pattern of intra-population variance the resulting evolutionary output in terms of differentiation between populations?

## Materials and Methods

### Samples

The study was based on wild-trapped populations of house mice (*Mus musculus* domesticus). Sampling included mainland populations (Southern France and Northern Italy) and insular populations from Sardinia, Corsica, and Piana, an islet 0.06 km^2^ large and 300 m off the Corsica coast close to Bonifacio. Variation within *Mus musculus domesticus* was further documented by samples from Iran, Denmark and Marion Island. A set of *Nannomys mattheyi* was further considered (Table 1).

**Table 1:** Geographic groups (with localities of trapping) documenting the variation within the house mouse *Mus musculus domesticus* (*M.m.d.*), and the additional sample documenting the pygmy mouse *Nannomys mattheyi*.

Species	Group	Region	Locality	Collection	UM1	LM1
*M.m.d.*	FR-GAR	Mainland France	Gardouch	CBGP	68	67
	FR-MTP	Mainland France	Montpellier and surroundings	ISEM	27	23
	IT	Mainland Italy	Lombardy & Emilia Romagna	ISEM	40	33
	CO-FAN	North West Corsica	Fango Valley	ISEM	53	51
	CO-S	South Corsica	Bonifacio	ISEM	9	10
			Bavella	MNHN	6	5
	Piana	Piana islet	Piana	ISEM	6	6
	SARD	Sardinia	Colline Romana	ISEM	11	11
*M.m.d.*	IRAN	Iran	Avhaz	ISEM	10	9
	DK	Jutland, Denmark	Egtved	ISEM	14	13
	MARION	Sub-antarctic	Marion Island	ISEM	12	11
*Nannomys mattheyi*			Mali	ISEM	7	7

Material housed in the collection of the Centre de Biologie et Gestion des Populations (CBGP, Baillarguet, France), of the Institut des Sciences de l'Evolution de Montpellier (ISEM, Montpellier, France) and of the Museum National d'Histoire Naturelle (MNHN, Paris, France). Number of teeth measured: UM1 (first upper molar); LM1: first lower molar.

### Size and shape descriptors

The overall shape of each molar was measured as the outline of the two-dimensional projection of the tooth viewed from the occlusal surface, with focus towards the basis of the crown at the widest part of the tooth. This outline registers the relative position and importance of the main cusps, as well as the presence of the unusual furrow at the forepart of the tooth that should correspond to an anterior elongation of the outline. The 2D projection further presents the advantage to be relatively invariant with the degree of wear of the molars in murine rodents [24]. The first upper and lower molars were measured. Depending on their state of preservation, either right or left molars were considered, the left ones being submitted to a mirror image in order to be measured as right ones.

For each tooth, 64 points at equally spaced intervals along the outline were sampled. Two main Fourier methods, with different advantages and drawbacks, can be applied to this data set. Any Fourier analysis describes the original outline by the variations of one or several parameters that are then approximated by a sum of trigonometric functions of decreasing wavelengths, the harmonics. The most commonly used method is the Elliptic Fourier transform (EFT) [25]. It is based on separate Fourier decompositions of the incremental changes along *x* and *y* as a function of the cumulative length along the outline. Any harmonic corresponds to four coefficients (FC): *A_n_* and *B_n_* for *x*, and *C_n_* and *D_n_* for *y*, defining an ellipse in the *xy*-plane. The coefficients of the first harmonic, describing the best-fitting ellipse to the original outline, are used to standardise the size, orientation, and starting point of the object. These standardisations constitute a major advantage of the EFT; yet, the Fourier coefficients are somehow redundant because the variations along *x* and *y* are related when considering a closed outline. An alternate method is a Radial Fourier Transform (RFT) that describes the original outlines as variations of the radius (distance from each point to the centre of gravity of the outline) that are then decomposed by a Fourier procedure. Any harmonics corresponds to two coefficients: *A_n_* and *B_n_*. The zero harmonic amplitude is proportional to the size of the outline and is used to standardise all FCs in order to retain shape information only [24].

Since the present study aimed to analyse patterns of (co-) variation, it seems preferable to minimise the measurement error and the number of variables. Hence, a combination of both methods (“REFT”) was used to optimise on the one hand, the standardisation of the outlines according to orientation and starting point, and on the other hand, a minimal number of variables [26]. EFT was applied to the 64 points of the outline, and a reconstructed outline of each tooth was obtained, the orientation being standardised according to the major axis of the first ellipse, and the starting point as the intersection of the outline with this major axis. The reconstructed outline was described by 64 points as the original, without loosing much detail in the outline since 16 harmonics were retained (i.e. 64 FCs for 64 initial points). RFT was then applied to the new 64 points, obtaining a set of Fourier coefficients standardised by size.

A characteristics of the Fourier analysis is that the higher the rank of the harmonic, the more details it describes. Hence, for simple shapes like murine molars, the contribution of the harmonics decreases as their rank increases whereas the amount of measurement error increases concomitantly (e.g. [26]). The seventh harmonic was chosen as a threshold optimizing the trade-off between a satisfying description of the tooth shape and the amount of measurement error. The shape of each tooth was thus described as a set of 14 FCs (2 coefficients of the REFT per 7 harmonics).

For comparison purpose with shape variables, the zeroth harmonic of the RFT was primarily considered as size estimator, because directly related to the Fourier coefficients as being used for standardized them by size. Additionally, the length each molar were automatically recorded during the extraction of the outline.

### Direction of variance, direction of co-variation between teeth, and allometry

Directions of evolution between populations were evaluated as the difference of the averaged FCs per locality. The main direction of shape variance was calculated as the first eigenvector (V1) of the variance-covariance (VCV) matrix of the FCs. Main directions of co-variation between the first upper and lower molars were estimated using Partial Least Squares (PLS) analyses [27]. This multivariate technique decomposes the matrix of co-variance between two sets of variables, here the sets of FCs of two different molars, into principal axes, one for each set of variables. PLS axes can be extracted from any set of characters; how much the covariation expressed by the PLS axes corresponds to a significant covariation between the characters can be evaluated by evaluating how much the scores on the PLS first axis of the first character are correlated with the scores on the PLS axis of the second character. Finally, the size – shape relationship was assessed by calculating directions of allometry by multiple regressions between size and FCs.

### Correlations between directions of shape changes

The correlations between vectors of differentiation and between directions of main variance were estimated by the angle between the two vectors ( = the arc cosine of the inner product of the two vectors elements). Simulation of angles between random vectors (vectors of 14 dimensions with random components) was used to assess the significance of such correlations between vectors [28], [29]. Fifty thousand simulations provided the following significance threshold for the absolute value of the inner product “*R*”: probability that the observed *R* is lower than observed between random vectors: *P*<0.01, *R* = 0.651; *P*<0.001, *R* = 0.770.

### Bootstrap procedures

The sampling of the initial populations may affect the evaluation of morphometric parameters such as mean shape and variance, and even more the estimation of directions of main variation and allometry [30], [31]. Hence, a bootstrap procedure was used to estimate the precision of each vector. Each group was bootstrapped 100 times. The eigenvectors of the VCV matrix, the allometric direction and the PLS directions between the UM1 and LM1 were calculated on these bootstrapped groups, and compared with the estimate based on the initial sample. The distribution of the bootstrapped directions around the original one was described by the mean coefficient of correlation and angle.

### Comparison between distance matrices

The degree of integration between UM1 and LM1 was estimated by how much a divergence of the first was finding a counterpart in the second. We evaluated inter-individual shape distances as Euclidean distances based on the FCs for the UM1 and the LM1. These distance matrices were compared using a Mantel t-test.

## Results and Discussion

### Directions of greatest variation are conserved between populations

The shape of each tooth was quantified using an analysis of the two-dimensional projection of its occlusal surface; this analysis provided a series of shape variables (fourteen Fourier coefficients, FCs) that were standardized by size. The first eigenvector (V1) of the variance-covariance (VCV) matrix based on these shape variables provides the expression of the main direction of variance in the space of the 14 FCs. In this multidimensional space, the direction of two vectors can be compared by computing the inner product of the two vector elements that provides an estimate of the correlation *R* between the two vectors. This value is the cosinus of the angle between the two vectors. Simulation of angles between random vectors was used to assess the significance of such correlations. The confidence in the estimation of each vector was assessed using a bootstrap procedure, comparing the original vector to 100 bootstrapped estimations.

Among our sample (Fig. 1), two populations of house mice had sufficient sample size to allow a reliable estimate of these directions: the mainland population of Gardouch (Southern France) and the Corsican population of the Fango valley. The directions of greatest variance were estimated with a high confidence (mean R between original vector and bootstrapped estimates >0.92). Directions were similar in Gardouch and Fango for the first upper molar (*|R|* = 0.869, *P_lower than random_*<0.001) and its lower counterpart (*|R|* = 0.901, *P_lower than random_*<0.001). These directions of greatest variance represented around 1/3 of the total variance (UM1 Gardouch: 39%; LM1 Gardouch: 36%; LM1 Fango 35%) but reached ½ of the total variance for UM1 in Fango (51%).

The high correlation between the directions of greatest variance in Gardouch and Fango argue for their conservation among populations. This is in agreement with other studies reporting a conservation of the major direction of phenotypic variance over a much longer evolutionary time-scale [29], [32], [33], [34]. Such stability over evolutionary time-scales was the prerequisite for a potential role of these directions as favoring the direction of evolution. This prerequisite being validated, we investigated whether the directions of greatest variance indeed channel evolution, by evaluating if they were correlated with evolutionary differences between populations.

### Directions of greatest variance as lines of least resistance to evolution

**Figure 2:**
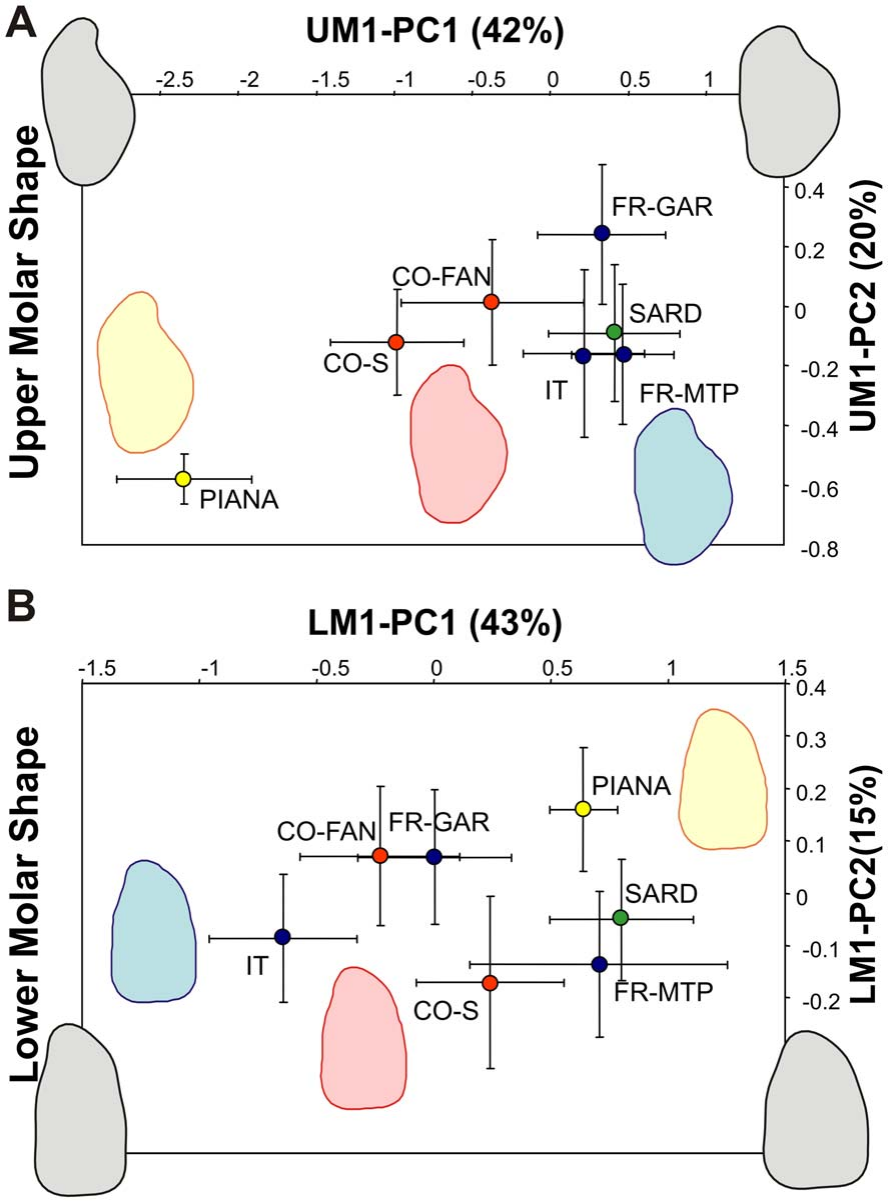
Inter-population shape differentiation of the first upper and lower molars. (A) Upper molars; (B) lower molars. Symbols are average values per geographic groups ± standard deviation. Shape axes correspond to the first two principal components describing variation of the Fourier coefficients of the outline analysis. PC1 corresponds to the V1 vector of the total sample. Shape changes along the first axes are visualized by reconstructed outlines corresponding to three times the unit variation along V1.

Directions of evolution were evaluated in two ways: first, as the first eigenvector of a VCV matrix including all samples, and hence expressing the total variance across mainland and the various islands. Despite including a part of intra-population variance, this direction is highly influenced by inter-population differences (correlation with the first eigenvector based on mean per populations only: UM1 |R| = 0.941, LM1 |R| = 0.995, *P*>0.999) and can be reliably estimated based on more than 200 specimens. This analysis also provides a visualization of the differentiation across the geographic coverage included in the present study (Fig. 2). Second, the direction of shape change between two populations of interest was evaluated as the difference between their mean FCs. Based on the pattern of differentiation (Fig. 2), it appears that populations from the mainland cluster as expected; they were thus pooled into a global mainland reference sample since the phylogeographic relationships between insular mice and their surrounding mainland relatives is unclear. Sardinian teeth were close to the mainland samples for both the first upper and lower molar. Corsican UM1 diverged markedly, UM1 from Piana being even more extreme along the first axis of total variance. In contrast, the differentiation between populations was much less marked for the LM1, only Piana emerging as slightly divergent from an otherwise unstructured variation pooling mainland, Corsican, and Sardinian samples.

Results regarding the first upper molar support the role of the direction of greatest variance as a line of least resistance to evolution. The direction of main variance in Gardouch is correlated to the total variance across the whole geographic coverage (|R| = 0.848, *P<0.001*) and to the direction from mainland to Corsica (|R| = 0.723, *P<0.001*). The correlation is even higher when considering the direction of main variance in the Corsican population of Fango (correlation with: the total variance |R| = 0.996, the shape change from mainland to Corsica |R| = 0.916, the shape change from Corsica to Piana |R| = 0.838; all *P<0.001*).

Similarly, the direction of greatest variance of the first lower molar is correlated with the total variance (V1Gardouch |R| = 0.964, V1Fango |R| = 0.961, *P<0.001*). These vectors are also correlated with the direction of the only marked morphological differentiation, namely from Corsica to Piana (V1Gardouch |R| = 0.819, V1Fango |R| = 0.787, *P<0.001*).

These results provide strong support for the role of the direction of greatest variance as a line of least evolutionary resistance. Developmental studies and results from quantitative genetics have strongly undermined the simplistic view of the genotype – phenotype relationship. Redundant genetic networks can lead to a similar phenotypic signature for different quantitative traits [35]; changes in regulatory regions can drive developmental pathways to produce dramatically different phenotypic outputs without much change at the genetic level [36], [37]; subtle genetic or epigenetic changes in the temporal dynamics of development can also lead to considerable phenotypic differences in genetically similar animals [6], [9], [10], [38]. Considering the phenotypic variance in wild populations integrates all these aspects that make a trait prone to vary. In agreement with the expectation that microevolution would surf on the genetic and developmental potentialities present within populations we found that inter-population differences in tooth shape occurred following these lines of least resistance.

### Upper and lower molars: an integrated system?

In the above analysis, we considered each tooth separately. Yet, occluding teeth are not expected to be free to vary independently: functional constraints at any step in their evolution should have molded a strong integration between these characters [39], [40]. This is especially true for the first molars of murine rodents, because the upper molar exclusively occludes with the first lower molar [41]. In agreement, we have previously evidenced a strong integration between occluding teeth in house mice using a quantitative comparison of upper and lower molar shape [26]. Here, we further evidenced this integration between the first upper molar and its occluding counterpart since the shape distances (Euclidean distances based on the FCs) based on the UM1 and the LM1 were correlated within each of the reference populations (Mantel test: Gardouch, 64 specimens, R = 0.171, *P_random Z.<obs. Z_* = 0.992; Fango, 51 specimens, R = 0.252, *P* = 0.999).

We next questioned whether the co-variation between occluding teeth would parallel the directions of greatest variance evidenced for each tooth, and thus if the co-variation between teeth was in agreement with the lines of least resistance predicted for each tooth. This was performed by estimating the main direction of co-variation between the UM1 and LM1, using the Partial Least Squares (PLS) method [27].

Both in Gardouch and in Fango, PLS axes were extracted which accounted for a significant covariation between UM1 and LM1 (correlation of UM1 PLS scores vs. LM1 scores: Gardouch, R = 0.501, P<0.001; Fango, R = 0.636, P<0.001). A prerequisite was to confirm that these directions of co-variation were stable between populations, i.e. that PLS Gardouch was correlated with PLS Fango (UM1 |R| = 0.937, LM1 |R| = 0.744, *P<0.001*). The next step was to compare these directions of co-variation to the directions of greatest variance for each tooth. In all cases, they appeared as highly correlated (PLS/V1 UM1 Gardouch |R| = 0.973, Fango |R| = 0.984; LM1 Gardouch |R| = 0.929, Fango |R| = 0.741; all *P<0.01*).

Hence, within each population, variations in shape of the first upper and lower molars appear as integrated, and the resulting direction of co-variation is conserved across populations and matches the directions of greatest variation of each tooth considered separately. Such a result is in agreement both with the expectations based on the functional constraints related to occlusion, and with the fact that a similar set of genes is required whichever tooth is considered [20], [42]. Hence, integration might result from sharing parts of the same genetic network and developmental pathways. Yet, despite the coherent pattern emerging within each population, the inter-population differentiation of the first upper and lower molars was not correlated (Mantel test between distance matrices: R = 0.128, *P_ random Z<obs. Z_* = 0.693). We interpret this apparent discrepancy as the result of a low variation of the LM1, resulting in no noticeable differentiation between populations and hence, with no clear pattern to be compared to the UM1. This would reconcile the overall integration of the two occluding teeth, and the apparent independence of their evolutionary patterns in the present study. Over a longer time allowing the LM1 to diverge as well, concerted evolution between UM1 and LM1 is expected and indeed observed in the murine fossil record (e.g. [43]).

### Differential allometry responsible for different evolvability of the upper and lower molars

We thus looked for a factor that may drive a faster evolution of the upper molar compared to the lower molar. Size evolution is often marked on islands, being part of the “island syndrome” [44], [45]. Accordingly, mice in Piana are larger and display larger teeth; the trend towards larger size is also present in Corsica although to a lesser degree. We therefore questioned if allometric variations might have participated to the evolutionary divergence. The relationship between tooth shape variables and tooth size was thus investigated using a multivariate regression of the FCs versus size. Indeed, we evidenced an important component of allometric variation within populations but for the UM1 only (UM1 Gardouch: *P* = 0.001, Fango *P*<0.001; LM1 Gardouch *P* = 0.039, Fango *P* = 0.144). The corresponding direction of variation was strongly correlated with the direction of greatest variance in Fango (|R| = 0.971) but not in Gardouch (|R| = 0.611), possibly because the more reduced range of size variation encountered in Gardouch hindered an estimation of the allometric variation as robust as in the Corsican population (robustness estimated by the dispersion of bootstrapped vectors around the vector computed on the whole population: Gardouch |R| = 0.878 smaller than Fango |R| = 0.963). Noteworthy, the direction of allometric variation within each population is also related to the directions of co-variation (Gardouch |R| = 0.711, Fango |R| = 0.935).

The occurrence of this allometric relationship driving the differentiation of the UM1 and not of the LM1, suggesting that a process related to size might have a larger impact on the upper molar than its lower counterpart. This might explain the higher evolvability of the UM1 in a case of insular evolution where a marked size increase is involved, despite highly integrated patterns of variation between upper and lower molars. Beyond the patterns of morphometric variation, we therefore attempted to question the developmental processes that might be at work in this intriguing differentiation.

### A developmental model for a size-related elongation of the first upper molar

**Figure 3:**
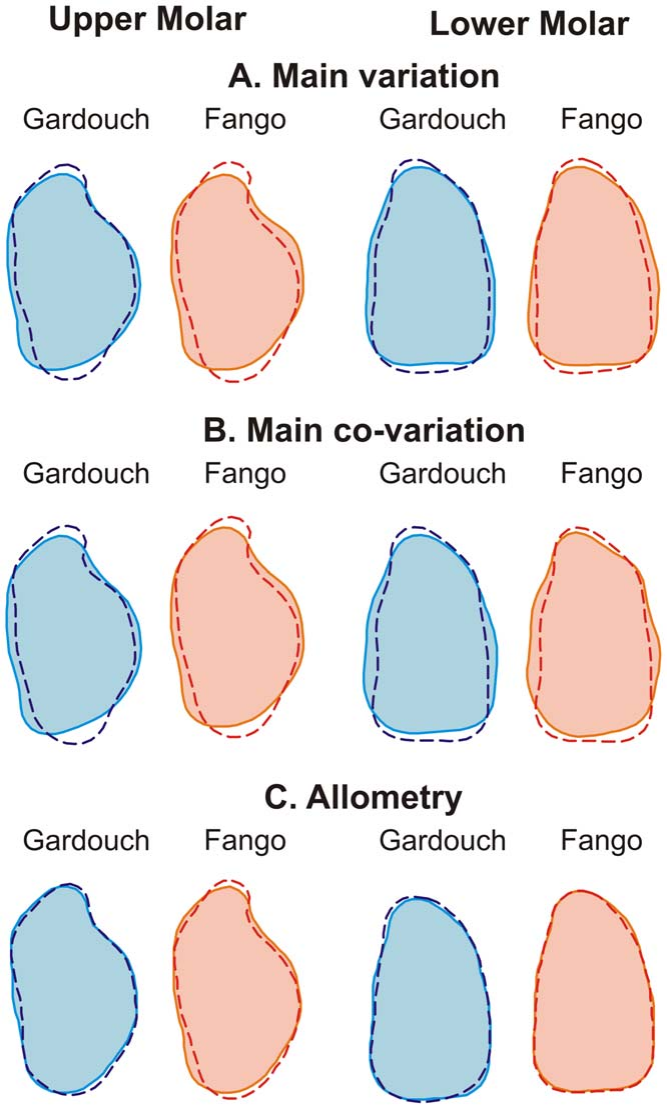
Main directions of shape changes in Gardouch (blue) and Fango (red). (A) main intra-population variation, (B) intra-population co-variation and (C) allometry. (A) Shape changes along the direction of greatest variance (V1) corresponding to ±3×V1. (B) Co-variation between the first upper and first lower molars based on Partial Least Squares analyses corresponding to ±0.03 PLS1. (C) Allometric change in molar shape corresponding to a 20% size increase, based on the multivariate regression between size and shape.

**Figure 4:**
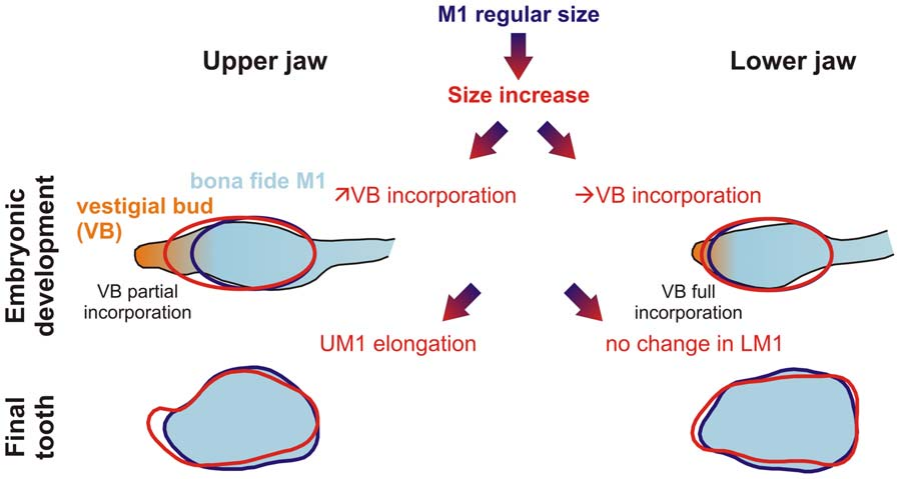
Schematic interpretation of the patterns of shape changes of the first upper and lower molars at the light of developmental processes. The processes during the development of the dental lamina are presented above, the zone corresponding to the M1 formation in blue and the anterior vestigial bud in orange. The putative phenotypic output on the final tooth shape is shown below. In both jaws, a vestigial bud, anterior to the first molar, aborts as the first molar forms. Later on, the signaling center of the first molar expands anteriorly, what determines incorporation of the vestigial bud into the first molar. A notable difference exists between the lower jaw (rapid abortion of the vestigial bud, complete incorporation in the LM1) and the upper jaw (later abortion, limited incorporation). We suggest that changes in incorporation might be responsible for the elongation of the UM1, and that these changes might be triggered by factors related to size increase, leading to an allometric elongation of the UM1. The process involved in the phenotypic difference between a tooth of regular size (in blue) and a large tooth (in red) is shown by a shaded blue-to-red arrow.

The directions of greatest variance and the main directions of co-variation, being parallel in the morphological space, correspond to highly similar shape changes on the molars that can be visualized by reconstructing theoretical outlines materializing the variation along each eigenvector (Fig. 3). The direction of greatest variance of both molars corresponds to an opposition between broad vs. slender molars, in Gardouch as in Fango. Such a pattern of intra-population variation seems to be a highly conserved feature in murine evolution since it has also been documented in the wild mouse *Apodemus sylvaticus* [26] that diverged from the house mouse more than 10 myrs ago [46]. It also characterizes the direction of greatest variance of the UM1 evidenced in successive fossil populations along an evolutionary trend where it constituted a line of least resistance to long term evolution of broader molars over 10 myrs [29]. This conserved pattern of greatest variation may constitute a potential to repeatedly evolve broad molars in evolutionary lineages of murine rodents [47]. The conservation over millions of years of this pattern of intra-population variation suggests that it may result from intrinsic developmental properties. The key aspect here is that the process should impact both tooth rows in a similar way, underlying thus the integrated variation between UM1 and LM1 (Fig. 4).

Yet, the pattern of greatest variance in Fango displays a characteristic feature related to the allometric relationship, namely an elongation of the forepart of the upper molar (Figs. 1, 2). This trend may be more marked in Fango due to the increase in tooth size in this population, whereas this variation is not expressed in the mainland population of Gardouch, characterized by much smaller teeth. The paralleling of this direction of greatest intra-population variance with the differentiation of the Piana population quantitatively evidence that the elongation of the tooth is achieved at an extreme degree in Piana, where a prestyle occurs anteriorly to the main cusps typical of the murine dental pattern (Fig. 1).

Developmental data obtained on laboratory mice may provide an insight into the basis of this morphological trend (Fig. 4). The development of vestigial buds, located anteriorly but close to the first molar precedes the development the molar row *per se* [48]. In the lower jaw, the vestigial bud is incorporated in the LM1's anterior part [49]. In contrast, in the upper jaw of the mouse, the vestigial bud persists longer and its incorporation in the UM1, if it occurs, is only partial [50]. Variations in the degree of incorporation of the vestigial bud may represent the developmental mechanism for the observed phenotypic variation of the UM1, allowing for a variable elongation of its forepart. This pattern seems to be favored by an increase in tooth size. We thus propose that the factors mediating size increase might also have an effect on the incorporation of the vestigial bud of the UM1. Since incorporation is complete for the LM1, the same developmental factors would only lead to an overall size increase without clear allometric shape changes of the LM1, providing an explanation for its lower inter-population differentiation.

**Figure 5:**
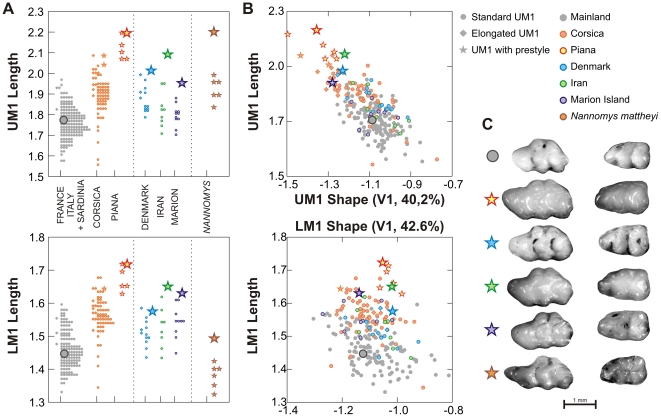
Size and shape of the first upper and lower molars in the mainland and Corsican sample and some populations displaying size increases: Iran, Denmark and Marion Island. A sample of the pygmy mouse *Nannomys mattheyi* is included. (A). Tooth length distribution per population, upper molar above and lower molar below. Large symbols correspond to teeth shown in C. (B). Size – shape relationship between these populations. Shape is estimated by a new synthetic axis including the additional populations. It is highly correlated to the total axis previously considered in the study (R = 0.999 for UM1 and LM1). Large UM1s are clearly shifted in shape, whereas this is not the case for the LM1. (C). Examples of UM1s displaying a prestyle in the different populations, with their lower counterpart.

Hence, the evolvability of the UM1 might be conditioned by intrinsic properties of the developmental system, allowing variation of the UM1 but not the LM1 even in response to similar triggering factors. Such a model has challenging consequences for evolutionary outputs. Marked morphological differences might evolve fast and repeatedly by following lines of least resistance that express intrinsic properties of the developmental system. Dissecting the developmental basis of these lines of least resistance is far beyond the scope of the present study, but we attempted to test our hypothetical model by investigating its expectations regarding the potential evolutionary outputs: an increase in tooth size should repeatedly trigger an elongation of the UM1. We thus gathered additional data on three wild populations from distant mainland geographic areas and one island known or suspected for a size increase [51]: Denmark, Iran, and the subantarctic Marion Island. Indeed, the three populations display teeth that are on average intermediate in size between our mainland populations and Corsica. In agreement with the expectations of our model, the largest of the upper teeth in these populations show an elongation of their forepart, even displaying the presence of a prestyle for the largest of them (Fig. 5). In contrast, the lower teeth do display no shift in shape despite being slightly larger as well (Fig. 5). Given the large geographic distance between these populations, these results document independent cases of parallel evolution. The prestyle can vary in its phenotypic expression from a discrete furrow to a cusp with a free apex, and our results suggest that it formed above a threshold value of around 1.9–2.0 millimeters in UM1 length (Fig. 5).

This direction of least resistance to evolution might be characteristics of the *Mus* genus, where some species display extreme morphologies of elongated UM1, especially in the subgenus *Nannomys* [52]. This may sound surprising since *Nannomys* are pygmy mice and thus, an elongation of their UM1 would be at the opposite of the expections of our model. Yet, for a tiny body size, *Nannomys* display extremely large molars. On a reduced sample of *Nannomys mattheyi*, we were able to document an average UM1 length close to 2.0 mm (Fig. 5). This is above the expected threshold observed in our house mouse sample for the occurrence of a prestyle, and indeed this species is characterized by an extreme elongation of the forepart of the UM1 and the occurrence of a prestyle.

These preliminary data point to the potential of evo-devo models to bridge the gap between micro- and macro-evolution. By pointing to a developmental process underlying lines of least resistance constituted by main directions of intra-population variation, it opens a challenging view regarding the interpretation of recurrent evolution of even strikingly divergent morphologies. We provide compelling evidence of independent evolution of derived morphologies of the first upper molar, related to contexts favoring a size increase. Cases of parallel evolution are often interpreted as a response to similar selective pressures but they can also be the result of developmental channeling [8], [53], as exemplified by our results. Lines of least resistance could indeed favor a response to selection along favored directions. Alternatively, however, our results suggest that recurrent evolution of a similar morphology might not systematically be the signature of parallel selection, but of evolution along lines of least resistance, possibly a side-effect of the selection for another trait (for instance a size increase on islands, e.g. [45]). Altogether with evidences of different evolvability of traits that are nevertheless integrated within populations, these results might unify apparently discrepant patterns of evolution in the dentition of the murine rodents.
